# Statistical properties of interval mapping methods on quantitative trait loci location: impact on QTL/eQTL analyses

**DOI:** 10.1186/1471-2156-13-29

**Published:** 2012-04-20

**Authors:** Xiaoqiang Wang, Hélène Gilbert, Carole Moreno, Olivier Filangi, Jean-Michel Elsen, Pascale Le Roy

**Affiliations:** 1 INRA, UMR 1348, PEGASE, 35042 Rennes cedex, France; 2 Agrocampus Ouest, UMR 1348, PEGASE, 35042 Rennes cedex, France; 3 INRA, UMR 1313, GABI, 78352 Jouy en Josas cedex, France; 4 INRA, UMR 444, LGC, 31326 Castanet Tolosan cedex, France; 5 INRA, UMR 631, SAGA, 31326 Castanet Tolosan cedex, France

**Keywords:** QTL, linkage analysis, QTL location, bias

## Abstract

**Background:**

Quantitative trait loci (QTL) detection on a huge amount of phenotypes, like eQTL detection on transcriptomic data, can be dramatically impaired by the statistical properties of interval mapping methods. One of these major outcomes is the high number of QTL detected at marker locations. The present study aims at identifying and specifying the sources of this bias, in particular in the case of analysis of data issued from outbred populations. Analytical developments were carried out in a backcross situation in order to specify the bias and to propose an algorithm to control it. The outbred population context was studied through simulated data sets in a wide range of situations.

The likelihood ratio test was firstly analyzed under the "one QTL" hypothesis in a backcross population. Designs of sib families were then simulated and analyzed using the QTL Map software. On the basis of the theoretical results in backcross, parameters such as the population size, the density of the genetic map, the QTL effect and the true location of the QTL, were taken into account under the "no QTL" and the "one QTL" hypotheses. A combination of two non parametric tests - the Kolmogorov-Smirnov test and the Mann-Whitney-Wilcoxon test - was used in order to identify the parameters that affected the bias and to specify how much they influenced the estimation of QTL location.

**Results:**

A theoretical expression of the bias of the estimated QTL location was obtained for a backcross type population. We demonstrated a common source of bias under the "no QTL" and the "one QTL" hypotheses and qualified the possible influence of several parameters. Simulation studies confirmed that the bias exists in outbred populations under both the hypotheses of "no QTL" and "one QTL" on a linkage group. The QTL location was systematically closer to marker locations than expected, particularly in the case of low QTL effect, small population size or low density of markers, i.e. designs with low power. Practical recommendations for experimental designs for QTL detection in outbred populations are given on the basis of this bias quantification. Furthermore, an original algorithm is proposed to adjust the location of a QTL, obtained with interval mapping, which co located with a marker.

**Conclusions:**

Therefore, one should be attentive when one QTL is mapped at the location of one marker, especially under low power conditions.

## Background

For the last decade, several studies have shown that a large proportion of QTL are mapped at the markers locations whenever linkage analysis is applied. As to what regards dataset analyses, this bias first raised doubts in Spelman *et al. *[1], who observed a large proportion of significant test statistics at marker location when looking for QTL in five milk production traits. Walling *et al. *[2] have described the influence of markers in constructing the confidence intervals of QTL location and questioned whether QTL location was biased towards the location of markers instead of its true position. By applying the regression coefficients on the markers as suggested by Whittaker *et al. *[3], Walling *et al. *[4] calculated the proportion of putative QTL located at marker positions in a backcross population. They have reported a systematic bias for the estimated QTL position under the null hypothesis of the test, i.e. the hypothesis of no QTL on the linkage group. Moreover, results from linear regression methods for QTL detection have been reported to behave the same way the results from maximum likelihood methods in interval mapping approaches do [5]. The simulation studies by Walling *et al. *[4] have confirmed that these two approaches have similar biases on the estimated QTL position in a backcross population.

These previous works have shown that a bias on the QTL location occurs when genetic linkage analysis for QTL mapping is used in a backcross population. However, little research has been devoted to establishing which parameters give rise to that bias. What is more, no study has investigated how it affects linkage analysis applied to outbred populations. In order to address these shortcomings, the present study aims at identifying the sources of that bias, in particular regarding the analysis of data issued from outbred populations.

This question is of critical importance in expression quantitative trait loci (eQTL) mapping. Indeed, a main objective is often to search for eQTL which co localize with QTL which influences agronomical performances. The accuracy of the eQTL locations is thus a fundamental element for experimental design optimization, especially since experimental designs for gene expression analyses are generally of moderate size due to the cost of phenotyping. The pioneer work of eQTL detection can be traced back to the emergence of the concept of genetical genomics [6]. During the past decade, QTL mapping was widely applied to the detection of eQTL, for example in yeast [7], mice [8], human [9,10], maize [11] and pig [12]. Generally, mapping procedures were used to map eQTL considering each transcript expression level as one quantitative trait in a trait by trait analysis.

**Figure 1:**
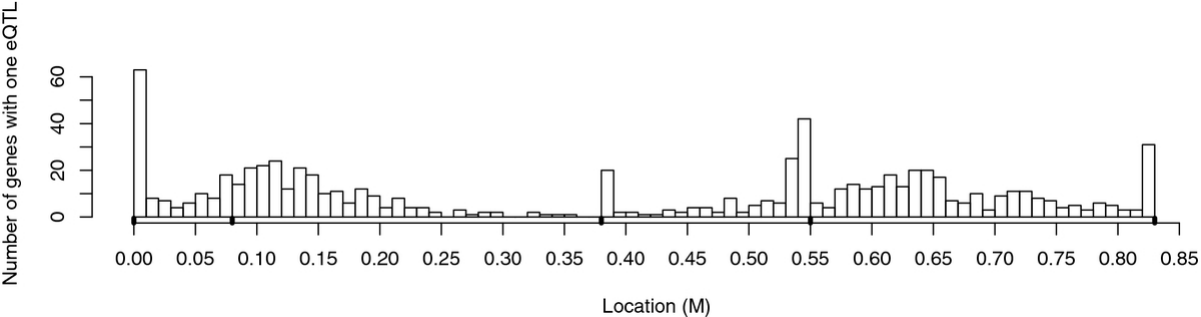
**eQTL mapping in pigs: example of distribution of the eQTL locations (chromosome 18)**. 756 eQTL were mapped on the chromosome 18 which have 5 microsatellite markers located at 0 M, 0.08 M, 0.39 M, 0.54 M and 0.83 M, respectively (black points on the *X *axis).

Recently, we have carried out linkage analyses by interval mapping on high throughput transcriptomic data from several familial QTL detection designs, in pig [13], in poultry [14] and in trout [15]. Because of the high dimensionality of the phenotypes, these eQTL analyses have highlighted to the bias of interval mapping estimation of the QTL location. We observed that the number of eQTL detected at marker locations was consistently higher than between marker locations: for instance, with the analysis of 6 665 gene expressions in a population of 325 pigs, we found 756 eQTL on the chromosome 18 distributed as shown in Figure 1. It appeared that the eQTL were significantly more often mapped on marker locations rather than between marker locations.

Hence, in order to qualify this possible bias on the estimated QTL location in outbred populations and to specify which parameters influence it, this paper presents a study of the QTL location accuracy. Firstly, in order to make things more concrete, we explored the empirical distribution of the LRT along the linkage group under the null hypothesis of "no QTL" on a real dataset. Secondly, analytical developments were carried out so as to identify the parameters which influence the QTL location accuracy. Since they are impossible to realize for outbred populations, because of the test statistic complexity, a more simple case of a backcross type population, i.e. a backcross between inbred lines, was considered at that stage. Thirdly, designs of outbred sib families were simulated and analyzed in order to characterize the bias variability under the null and the alternative ("one QTL segregating on the linkage group") hypotheses. Such parameters as population size and marker density under the null hypothesis, as well as QTL effect and simulated QTL location under the alternative hypothesis were taken into account. Finally, an approach as to how to adjust the QTL location estimation, for a QTL located at the position of one marker, was suggested.

## Results

### The LRT distribution along the linkage group under the null hypothesis

**Figure 2:**
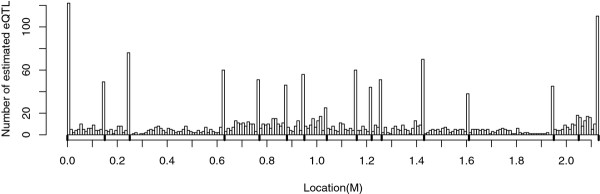
**QTL mapping in pigs: empirical distribution of the estimated QTL location under H0 (chromosome 1)**. Results are based on 2000 simulations in a population of 4 sires and 325 offspring. There are 16 markers on the chromosome 1 (black points in the X axis).

**Figure 3:**
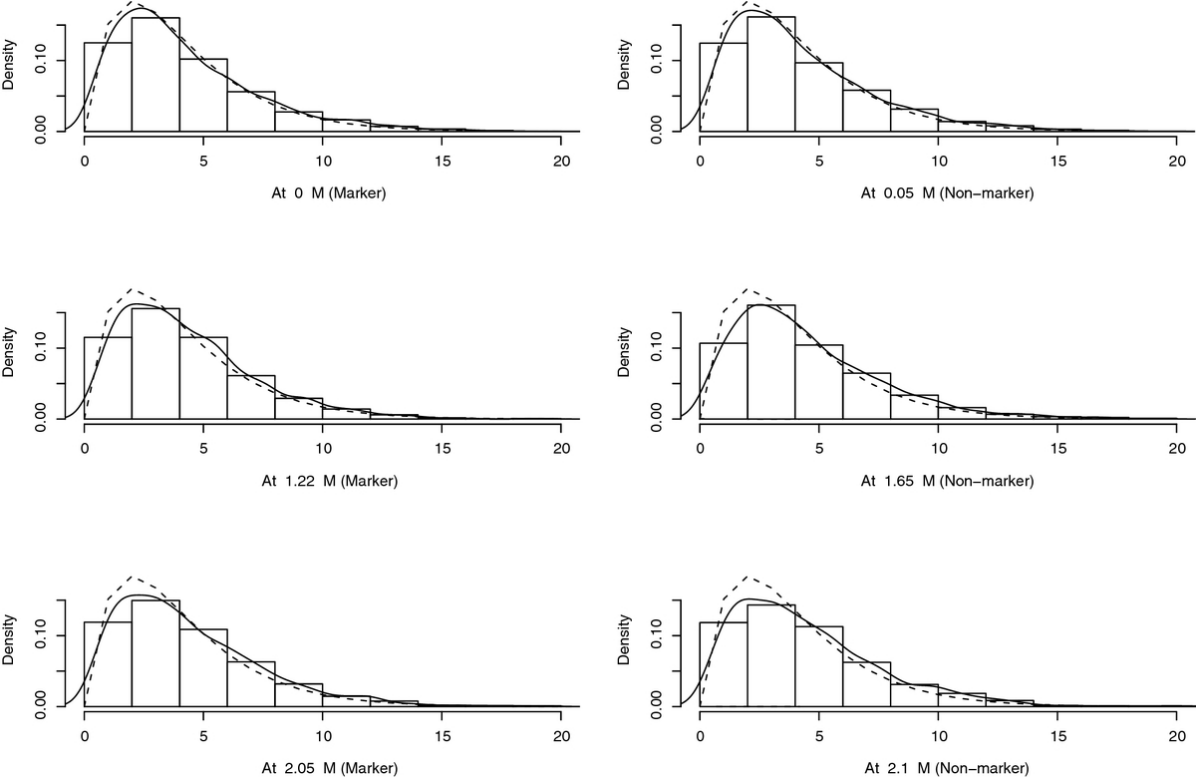
**QTL mapping in pigs: empirical distribution of the LRT at various locations**. Results are based on 2000 simulations in a population of 4 sires and 325 offspring. The line represents the density obtained with a Kernel method, the dotted line represents the density of a χ^2 ^with 4 d.f.

**Figure 4:**
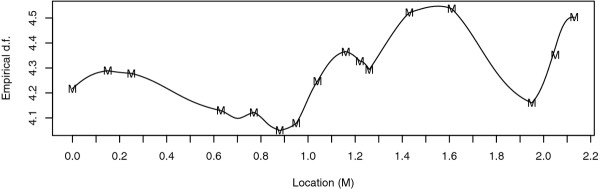
**QTL mapping in pigs: empirical degrees of freedom of the χ^2 ^distribution**. M indicates the locations of the markers.

By using the real pedigree and genotypes structure from an experimental design in pig, 2 000 simulations of phenotypes under the null hypothesis of "no QTL on the linkage group" were performed. The distribution of the estimated QTL location, i.e. the location of the maximum LRT, was obtained (Figure 2) on the chromosome SSC1 which carried 16 microsatellite markers (black points in the X axis). It clearly shows that a large proportion of QTL was found at a marker location. The histograms in Figure 3 show the empirical distributions of the 2000 LRT at some locations on the chromosome, both markers and non-markers. The LRT at each location followed a *x^2 ^*distribution, with degrees of freedom ranging from 4.05 to 4.55, according to a Kolmogorov-Smirnov (KS) test at *α *= 0.01. This was generalized to all tested positions on the linkage group in Figure 4, where the distributions of the LRT characterized by the number of degrees of freedom under the null hypothesis at markers was not very different from the distributions obtained for positions between markers.

**Figure 5:**
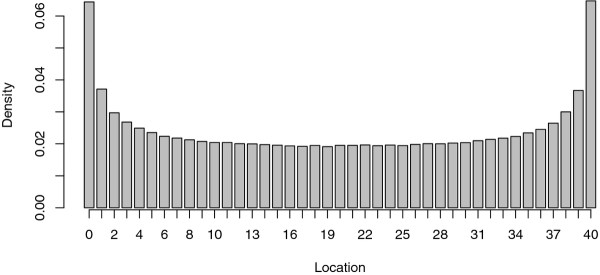
**Empirical distribution of the maximum of the Ornstein-Uhlenbeck process**. Results are based on 1 million of simulations.

It should be noted (Figure 2) that the proportion of QTL locations estimated at the two extreme marker positions of the linkage group was higher than for the other markers under the "no QTL" hypothesis. The asymptotic distribution of the LRT process at marker positions is known to be the square of an Ornstein-Uhlenbeck (OU) process as pointed out by Lander and Botstein [16] and proved by Cierco [17]. As indicated in Rabier *et al. *[18], when test statistics are performed only on markers, the OU process follows an autoregressive process of order 1. After 1 million of simulations for this process, we found that the probability for the maximum of the OU process to be on the bounds is higher than within the interval (Figure 5). This property of the OU process was consistent with the fact that we observed a large proportion of QTL localised at the extreme marker positions in comparison with the other markers.

### The QTL location bias expression in a backcross population

In order to investigate the bias on the QTL location under the hypothesis of "one QTL", we considered a linkage group limited to an interval [0,*T*] between two markers *M*_1 _(alleles *M*_1 _and *m*_1_) at 0 and *M*_2 _(alleles *M*_2 _and *m*_2_) at *T *flanking a QTL (alleles *Q *and *q*) in a backcross population obtained from the cross *M*_1_*M*_1_*QQM*_2_*M*_2 _× *M*_1_*m*_1_*QqM*_2_*m*_2_.

Let *y_k _*be the phenotypic value for the individual *k *= 1,...,*n*. Assuming a QTL located at the location *t_0 _*, the genetic model for *y_k _*is:

(1) $$y_{k}=\mu +\frac{a}{2}g_{k}\left(t_{0}\right)+e_{k},$$

where *μ *denotes the overall mean, *a *denotes the QTL allelic substitution effect, *g_k_*(*t_0_*) is the genotypic value of *k *at the QTL position, which takes 1 or -1 value depending on the QTL allele, *Q *or *q *respectively, received by *k *from its heterozygous parent and *e_k _*is a random normal variable with mean 0 and variance σ^2^. In order to simplify calculations, we set *μ *= 0 and σ^2 ^= 1 and used a linearized likelihood function instead of a mixture of two normal distributions (e.g. [19]). In this case, the interval mapping method is the same as the regression method for QTL detection, and the model (1) is modified as follows:

(2) $$y_{k}=\frac{a}{2}x_{k}\left(t_{0}\right)+e_{k},$$

where *x_k_*(*t*_0_) = **E**[*g_k_*(*t*_0_)|*M^k^*] and *M^k ^*denotes the genotype of the individual *k *at markers *M_1 _*and *M_2_*. Let *θ_t-t' _*be the recombination rate in the distance |*t *- *t*'|, then for all *k *(see Appendix I):

$$\begin{array}{ccc} x_{k}\left(t\right)= & \left\{\begin{array}{cc} \left(1-\theta_{t}-\theta_{T-t}\right)/\left(1-\theta_{T}\right) & \text{if}\;M^{k}=M_{1}M_{1}M_{2}M_{2}\\ \left(\theta_{T-t}-\theta_{t}\right)/\theta_{T} & \text{if}\;M^{k}=M_{1}M_{1}M_{2}m_{2}\\ \left(\theta_{t}+\theta_{T-t}-1\right)/\left(1-\theta_{T}\right) & \text{if}\;M^{k}=M_{1}m_{1}M_{2}m_{2}\\ \left(\theta_{T-t}-\theta_{t}\right)/\theta_{T} & \text{if}\;M^{k}=M_{1}m_{1}M_{2}M_{2} \end{array}\right) & \end{array}$$

and the LRT at the position *t *is calculated as

(3) $$LRT\left(t\right)=\frac{{\left[\sum y_{k}\;x_{k}\left(t\right)\right]}^{2}}{\sum x_{k}^{2}\left(t\right)}.$$

Note that given a position *t, {x_k_*(*t*)*}_k = 1, ..., n _*is a sequence of *i.i.d*. random variables with the same expected value 0 and the same variance (see Appendix III), noted *Var(x(t))*. Hence, if we replace *y_k _*by the model (2) in equation (3), then the *LRT(t) *consists of three terms as (see Appendix II):

$$LRT\left(t\right)=f\left(t\right)+\varepsilon_{1}\left(t\right)+\varepsilon_{2}\left(t\right),$$

where $f\left(t\right)=\frac{1}{4}a^{2}{\left[\sum x_{k}\left(t_{0}\right)x_{k}\left(t\right)\right]}^{2}/\sum x_{k}^{2}\left(t\right)$ is a function of variable *t*, the noise *ε*_1_(*t*) is the LRT at *t *under the no QTL hypothesis and the noise *ε*_2_(*t*) follows approximately a normal distribution with mean 0 and variance $na^{2}Var\left(x\left(t\right)\right)\rho_{tt_{0}}^{2}.$ Note that:

$$\arg\underset{t\in \left[0,T\right]}{\max}LRT\left(t\right)=\arg\underset{t\in \left[0,T\right]}{\max}\frac{1}{n}LRT\left(t\right),$$

i.e. the bias on *LRT(t) *behaves similarly to the bias on *LRT*(*t*)/*n*. So this property allows to analyze the source of the bias in *LRT*(*t*)/*n *instead of *LRT(t)*. Using the law of large numbers, we have this following decomposition of *LRT *(*t*)/*n*:

(4) $$\frac{1}{n}LRT\left(t\right)\approx \frac{1}{4}a^{2}Var\left(x\left(t_{0}\right)\right)\rho_{tt_{0}}^{2}+\frac{1}{n}\varepsilon_{1}\left(t\right)+\frac{1}{n}\varepsilon_{2}\left(t\right),$$

It can be seen from formula (4) that the first term reaches its maximum at *t*_0 _since $\rho_{tt_{0}}\to 1$ when *t*→ *t*_0_. As seen in the previous section, the second term, which is proportional to the LRT at *t *under the "no QTL" hypothesis, reaches its maximum at the position of markers more often than at the positions between markers. As a result, the estimated QTL location will be biased towards the position of markers. However, when *n *or *a^2 ^*increase, or when *T *decreases, or when *t*_0 _approaches one marker location (see Appendix III), the deviation between the two first terms in formula (4) increases and the influence of the second term is reduced. Therefore, in our simple backcross population model, under the hypothesis of one QTL, when the population size, marker density, QTL effect increase or when the true QTL location approaches the position of a marker, the bias of the estimated QTL location is expected to be reduced.

### Simulations under H0

According to the preceding results, the estimated QTL location cannot be expected to be uniformly distributed on the chromosome under the null hypothesis of no QTL. Familial designs were simulated to test the influence of the population size and the marker density on this bias in outbred populations.

#### Impact of the population size

**Figure 6:**
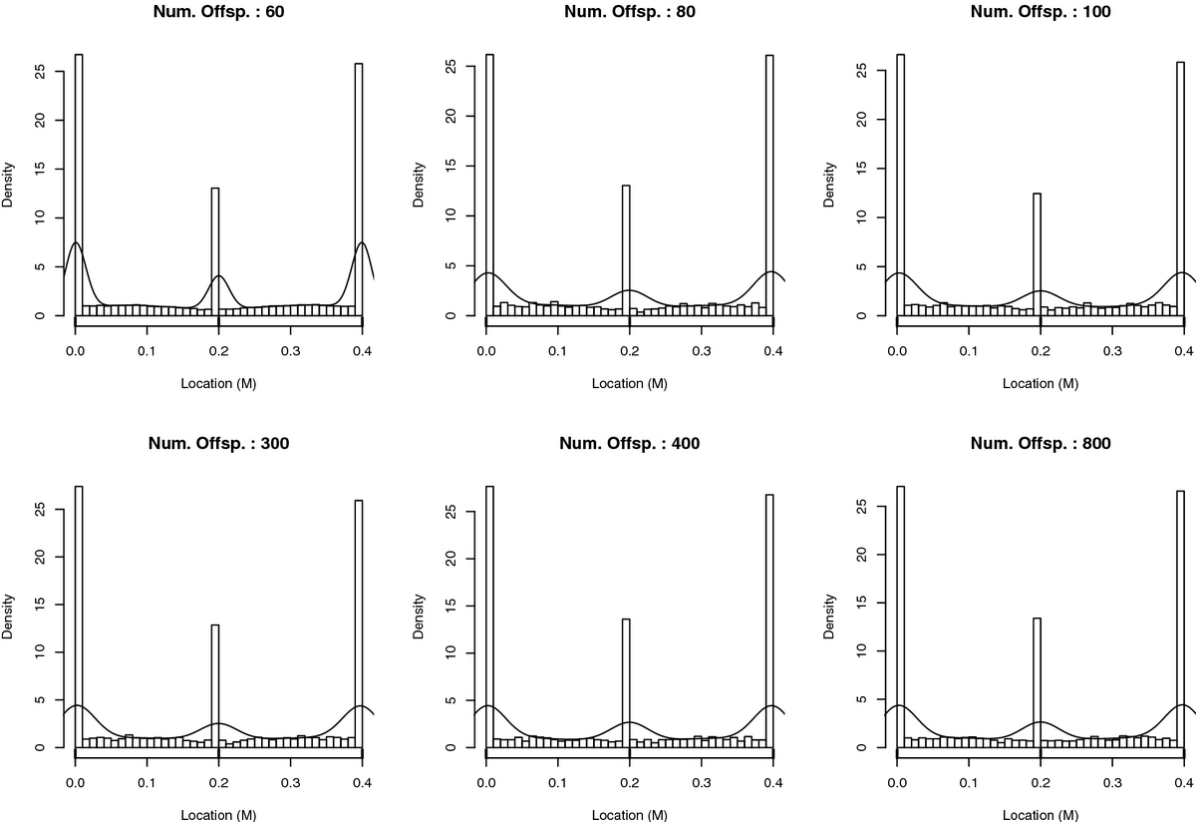
**Empirical distribution of the estimated QTL location according to the population size under H0**. Num. Offsp. indicates the number of offspring in the outbred population. Results are based on 5000 simulations per case. There were three markers at 0 M, 0.2 M and 0.4 M. The line represents the density obtained with a Kernel method.

Six different population sizes, from 60 to 800 progeny, were simulated with three markers located at 0 M, 0.2 M and 0.4 M on a 0.4 M linkage group. The empirical distributions of the estimated QTL location, obtained from 5 000 simulations for each population size, showed that the probability of mapping a QTL at a marker location was always higher than that of mapping it between markers (Figure 6). As shown in Table 1, the proportion of QTL which co-localized with the markers looked independent from the population size. When comparing the estimated QTL location distributions, which were obtained with the different population sizes, using the Kolmogorov-Smirnov test, large p-values were obtained (> 0.97). This indicates that the population size did not influence the distribution of the estimated QTL location in a significative way under the null hypothesis.

**Table 1:** Proportion of estimated QTL locations at marker locations according to the population size under H0

	Number of individuals (*s *× *d *× *p*)^1^				
	
	60 (3 × 1 × 20)	100 (5 × 1 × 20)	300 (5 × 2 × 30)	400 (5 × 2 × 40)	800 (5 × 4 × 40)
Proportion^2 ^(%)	64.2	63.8	65.3	67.1	66.0

^1^The population structure was a mixture of full and half sib families for given numbers of sires (*s*), dams per sire (*d*), and progeny per dam (*p*).

^2^Proportion of QTL located at a marker position (3 markers at 0 M, 0.2 M and 0.4 M).

#### Impact of the marker density

**Figure 7:**
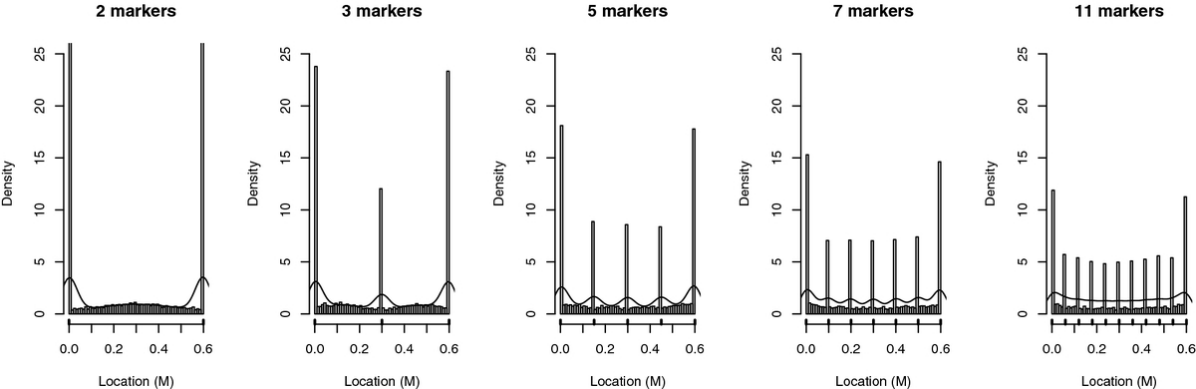
**Empirical distribution of the estimated QTL location according to the marker density under H0**. Results are based on 5000 simulations per case of in an outbred population of 300 individuals. The number of markers varied from 2 to 11 across along a linkage group of 0.6 M. The line represents the density obtained with a Kernel method.

The empirical distributions of estimated QTL locations were compared for five marker, with 2, 3, 5, 7 and 11 markers equally distributed on a 0.6 M linkage group (Figure 7). The bias towards the marker positions was systematic whichever the marker density. Except for 11 markers, the number of markers had little influence on the proportion of QTL located at a marker position (Table 2), i.e. the bias seemed to divide up between markers. However, this criteria was difficult to interpret because the number of markers was different in each of the cases studied. Moreover, in all cases, the two extreme markers concentrated more false locations than intermediate markers did. Since the number of markers was different, the distributions based on the marker density were expected to be different as well. The KS test was thus not suitable to test the influence of the marker density.

**Table 2:** Proportion of estimated QTL locations at marker locations according to the marker density under H0

	Number of markers^1^				
	
	2	3	5	7	11
Proportion^2 ^(%)	58.6	58.5	52.6	57.8	69.5

^1^The markers were evenly distributed on a linkage group of 0.6 M.

^2^Proportion of QTL located at a marker location for a population of 300 individuals.

### Simulations under H1

According to the analytical results obtained in a backcross type population, the population size and the marker density, as well as the QTL effect and location, were parameters which were very likely to influence the bias on the estimated QTL location under the alternative hypothesis.

#### Impact of the population size

**Figure 8:**
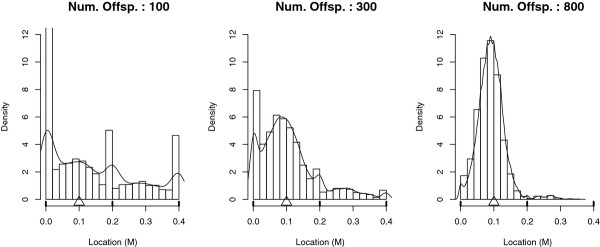
**Empirical distribution of the estimated QTL location according to the population size under H1**. Num. Offsp. indicates the number of offspring in the outbred population. Results are based on 5000 simulations per case. There were three markers at 0 M, 0.2 M and 0.4 M. Δ indicate the true QTL location. The line represents the density obtained with a Kernel method.

Three sizes of population were simulated: 100, 300 or 800 progeny. The empirical distributions of the estimated QTL location are given in Figure 8. One QTL was simulated at the 0.1 M location (Δ on the X axis) on a 0.4 M linkage group with three markers located at 0 M, 0.2 M and 0.4 M. The Figure 8 clearly shows a variability of the bias of the QTL location depending on the population size. It can be seen from Table 3 that the proportion of putative QTL that co-localized with a marker and the root mean square error (RMSE) of the QTL location became smaller when the population size increased. Table 3 also shows that the bias was correlated to the power of the analysis. However, considering only very significant LRT (*α *= 0.01), significant (*α *= 0.05) or all LRT (*α *= -), led to very similar values of RMSE or of proportion of QTL mapped on a marker location. The KS test indicated that the distributions of the estimated QTL position were significantly different depending on the size of the population studied (*p - values *= 0). Moreover, the Mann-Whitney-Wilcoxon (MWW) test showed that, when the population size increased, the median of the error of the estimated QTL position significantly decreased (*p - values *< 2.2e - 16).

**Table 3:** Power, RMSE of the QTL location and proportion of estimated QTL locations at marker locations according to the population size under H1

		Number of individuals (*s *× *d *× *p*)^1^		
		
	*α*^2^	100 (5 × 1 × 20)	300 (5 × 2 × 30)	800 (5 × 4 × 40)
Power^3 ^(%)	0.05	39	93	100
	0.01	15	81	100

RMSE^4 ^(cM)	-	13.9	8.7	4.2
	0.05	12.2	8.4	4.2
	0.01	11.4	8.0	4.2

Proportion^5 ^(%)	-	39.9	15.2	1.6
	0.05	29.7	14.0	1.6
	0.01	26.8	12.9	1.6

^1^The population structure was a mixture of full and half sib families for given numbers of sires (*s*), dams per sire (*d*), and progeny per dam (*p*).

^2^Significance level for the LRT.

^3^Power of the QTL detection at significance level *α*.

^4^RMSE of the estimated QTL location, the true QTL being located at 0.1 M on a 0.4 M linkage group.

^5^Proportion of QTL located at a marker location (3 markers at 0 M, 0.2 M and 0.4 M).

#### Impact of the marker density

**Figure 9:**
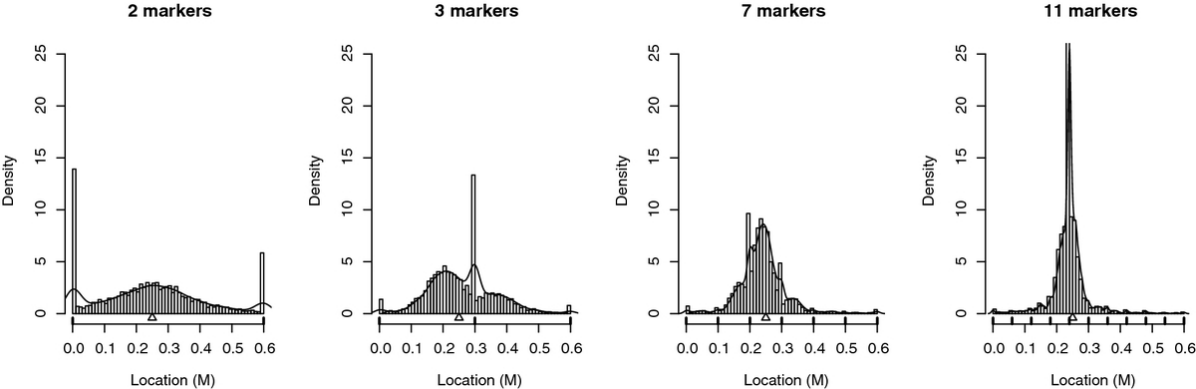
**Empirical distribution of the estimated QTL location according to the marker density under H1**. Results are based on 5000 simulations per case of an outbred population of 300 individuals. The number of markers varied from 2 to 11 along a linkage group of 0.6 M. Δ indicates the true QTL location. The line represents the density obtained with a Kernel method.

Figure 9 reports the QTL location distribution for a marker density from 2 to 11 markers equally distributed on a 0.6 M interval. One QTL was simulated at 0.25 M. It shows the advantage of using a high marker density in the QTL detection: when the marker spacing was minimal (i.e. 6 cM), the location of the QTL was very accurate. Table 4 shows that, when the density increased, the RMSE of the estimated QTL location decreased. So the QTL location was more accurately estimated. The dependency between the power of the analysis and the bias extent was confirmed. On the contrary, there were low variations of RMSE or proportion of QTL mapped at marker location according to α. Finally, as under H0, these tendencies were confirmed for the proportion of QTL located at a marker location, except for 11 markers.

**Table 4:** Power, RMSE of the QTL location and proportion of estimated QTL locations at marker locations according to the marker density under H1

		Number of markers^1^				
		
	*α*^2^	2	3	5	7	11
Power^3 ^(%)	0.05	62.2	91.6	94.4	96.5	97.5
	0.01	37.2	79.0	85.2	88.9	91.8

RMSE^4 ^(cM)	-	16.6	10.5	8.8	7.4	5.7
	0.05	14.9	10.1	8.4	7.2	5.6
	0.01	14.1	9.7	8.0	6.9	5.2

Proportion^5 ^(%)	-	19.0	15.4	12.5	11.8	35.0
	0.05	13.0	14.6	11.5	11.5	34.5
	0.01	10.8	13.7	10.7	10.5	34.0

^1^The markers were evenly located in a linkage group of 0.6 M.

^2^Significance level for the LRT.

^3^Power of the QTL detection at significance level *α *in a population of 300 progeny.

^4^RMSE of the estimated QTL location, the true QTL being located at 0.07 M on a 0.6 M linkage group.

^5^Proportion of QTL located at a marker location.

#### Impact of the QTL effect

**Figure 10:**
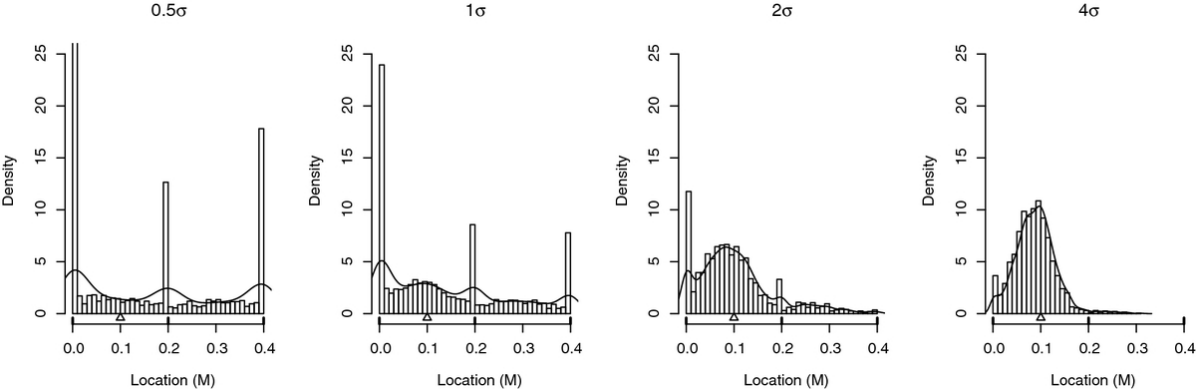
**Empirical distribution of the estimated QTL location according to the QTL effect under H1**. Results are based on 5000 simulations per case in an outbred population of 100 progeny. There were three markers at 0 M, 0.2 M and 0.4 M. The QTL effect varied from 0.5 *σ *to 4*σ*. Δ indicates the true QTL location. The line represents the density obtained with a Kernel method.

The empirical distributions of the estimated QTL location when the QTL effect increased from 0.5 phenotypic standard deviation (*σ*) to 4*σ *is shown in Figure 10. One QTL was simulated at 0.1 M on a 0.4 M linkage group with three markers equally spaced. The power of the QTL detection, the RMSE of the estimated QTL location and the proportion of estimated QTL locations at a marker are given in Table 5. Results indicated that, whenever the QTL effect increased, the bias decreased. As seen previously, the bias decreased when the power increased but the RMSE or the proportion of QTL mapped on a marker position were only slightly dependent on the test level. The KS test indicated that the distribution of the estimated QTL position was significantly different when the QTL effect changed (*p - values *< 2.2e - 16). The MWW test showed that, when the QTL effect increased, the median of the error of the estimated QTL position decreased (*p - values *< 2.2e - 16).

**Table 5:** Power, RMSE of the QTL location and proportion of estimated QTL locations at marker locations according to the QTL effect under H1

		QTL effect				
		
	*α*^1^	0.5 *σ*	1 *σ*	1.5 *σ*	2 *σ*	4 *σ*
Power^2 ^(%)	0.05	10.5	41.8	81.0	97.4	100
	
	0.01	2.9	19.5	58.1	89.8	100

RMSE^3 ^(cM)	-	17.0	13.6	10.3	8.0	4.4
	
	0.05	15.6	11.9	9.8	7.8	4.4
	
	0.01	15.4	10.9	9.1	7.6	4.4

Proportion^4 ^(%)	-	56.6	37.9	24.6	13.6	2.4
	
	0.05	37.1	28.8	22.2	13.2	2.4
	
	0.01	31.0	24.6	20.3	12.9	2.4

^1^Significance level for the LRT.

^2^Power of the QTL detection for significance level *α*.

^3^RMSE of the estimated QTL location in a population of 100 progeny with a true QTL located at 0.1 M.

^4^Proportion of QTL located at a marker location (3 markers at 0 M, 0.2 M and 0.4 M).

### Impact of the true QTL location

**Figure 11:**
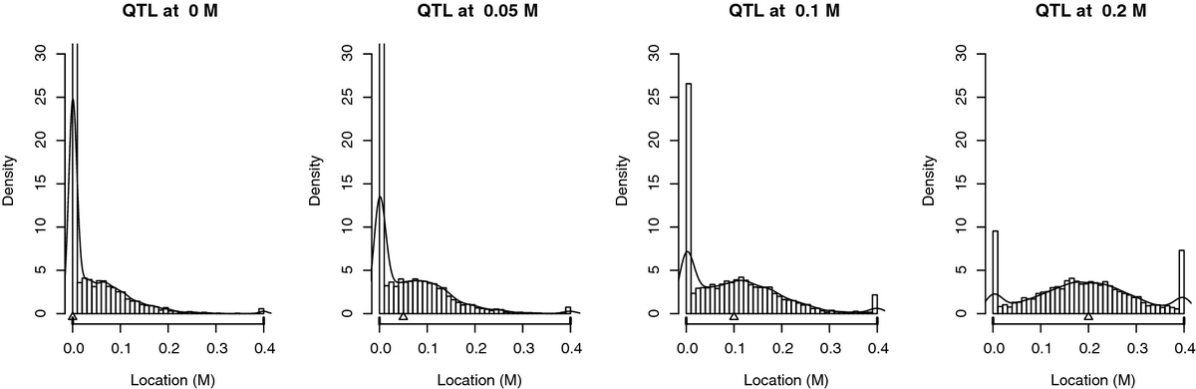
**Empirical distribution of the estimated QTL location according to the true QTL location under H1**. Results are based on 5000 simulations per case of an outbred population of 300 progeny. There were two flanking markers at 0 M and 0.4 M. The true QTL location varied from 0 M to 0.2 M. Δ indicates the true QTL location. The line represents the density obtained with a Kernel method.

Figure 11 shows the variation in the distribution of the estimated QTL location when the simulated QTL position (Δ on the X axis) moved towards the middle of two flanking markers on a 0.4 M linkage group. Table 6 shows the power of the QTL detection, the RMSE of the estimated QTL location and the proportion of estimated QTL positions at a marker position when the true QTL location changed from 0 M to 0.2 M. When the true QTL location tended towards the flanking markers, the power went up and the proportion of QTL locations at a marker location increased. On the contrary, the RMSE went down. The KS test confirmed the difference between the distributions of the estimated QTL location when the true QTL location varied (*p - values *< 2.2e - 16). The MWW test, which compared the medians of error of the estimated QTL location, confirmed the increase in accuracy when the true QTL location tended towards a marker location.

**Table 6:** Power, RMSE of the QTL location and proportion of estimated QTL locations at marker locations according to the true QTL location under H1

		QTL location (M)				
		
	*α*^1^	0	0.05	0.1	0.15	0.2
Power^2 ^(%)	0.05	96.1	92.3	87.2	82.0	81.2
	0.01	87.4	80.1	69.7	61.9	60.2

RMSE^3 ^(cM)	-	7.5	7.6	9.5	10.7	11.2
	0.05	7.2	7.2	9.0	10.1	10.7
	0.01	7.0	7.0	8.6	9.7	10.3

Proportion^4 ^(%)	-	51.5	38.6	26.6	19.0	16.2
	0.05	51.3	37.8	24.8	15.8	13.6
	0.01	50.9	37.2	24.3	13.8	11.2

^1^Significance level for the LRT.

^2^Power of the QTL detection for significance level *α*.

^3^RMSE of the estimated QTL location in a population of 300 progeny with a true QTL located at 0.1 M on a 0.4 M linkage group.

^4^Proportion of QTL located at a marker location (2 markers located at 0 M and 0.4 M).

### An algorithm to adjust the location of QTL mapped on markers

Analytical developments in backcross type population and simulation study in outbred type population demonstrated that the estimated position of the QTL is biased towards marker location under some circumstances. On the other hand, the decomposition of the LRT according to the formula (4) allowed to identify a putative cause of this bias: the residual error *ε*_1 _in the LRT both under "no QTL" and the "one QTL" hypotheses. Indeed, according to the decomposition of the LRT in the formula (4), if the QTL is not estimated at its true location, two residual errors may have generated the bias: *ε*_1 _and *ε*_2_. When the estimated QTL position is at a marker location, argmax_*t *_*ε*_2_(*t*) has a uniform distribution but argmax_*t *_*ε*_1_(*t*) is more often estimated at a marker location than between markers. In such a situation, *ε*_1 _is very likely to play a dominant role in the bias. On the contrary, when the estimated QTL location is not at a marker location, argmax_*t *_*ε*_1_(*t*) and argmax_*t *_*ε*_2_(*t*) are unknown for a given argmax_*t*_[*ε*_1_(*t*) + *ε*_2_(*t*)] error. Under these circumstances, it is impossible to predict the relative influence of *ε*_1 _and *ε*_2 _on the bias. On the basis of this observation, we propose an approach to describe the *ε*_1_(*t*) process and, consequently, adjust the estimated QTL position when a QTL co localizes with one marker, i.e. an approach to correct the "marker effect" on the bias of the estimated QTL location.

1. Obtain the vector which contains the LRT profile along the linkage group, calculated on the phenotypic data, say **L**_0_. **L**_0 _is maximum at the location of the marker *M*.

2. Under the "no QTL" hypothesis, simulate phenotypes and obtain LRT profiles until to have 1000 profiles which have their maximum at the position of the marker *M*, say *{***L_*i*_**}_*i *= 1,...,1000_

3. Calculate the 1 000 vectors V_*i *_= **L**_0_-**L**_i_,*i ϵ *1,...,1000.

4. Retain the 1000 locations where *{***V_*i*_***}*_*i *= 1, ..., 1000 _is maximum.

5. Obtain the adjusted position of the QTL as the mean of these positions:

$$\hat{P}=\text{Mean}\left\{p_{i}\;|\;p_{i}=\mathrm{argmax}V_{i},i=1,\ldots ,1000\right\}.$$

**Figure 12:**
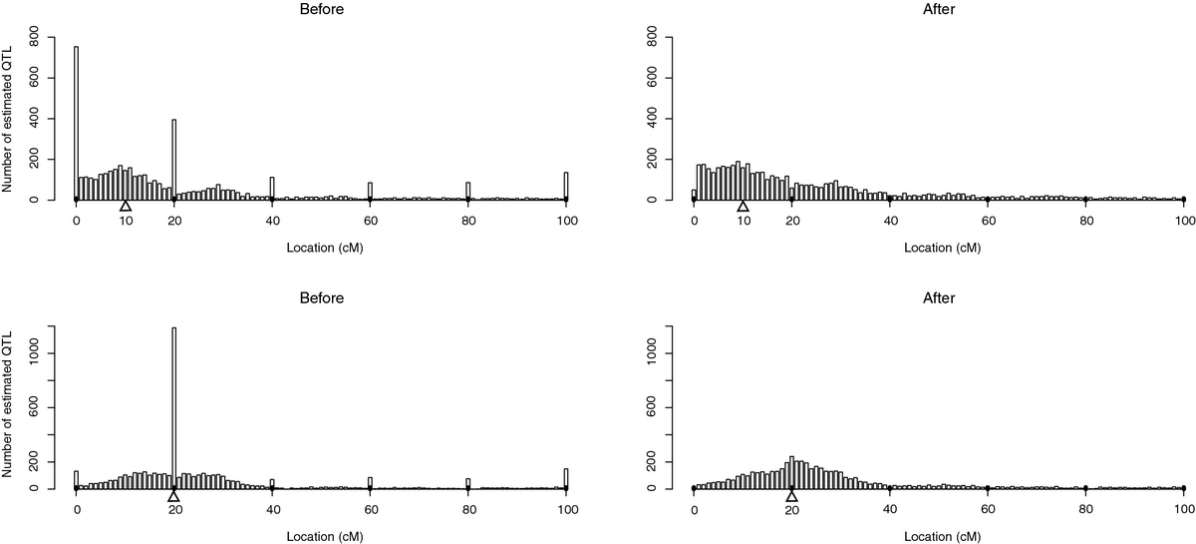
**Empirical distribution of the estimated QTL locations before and after adjustment**. Results are based on 5000 simulations per case in a backcross population of 100 progeny. There were six markers equally distributed on a linkage group of 1 M. The true QTL location indicated as Δ was set at 0.1 M or 0.2 M.

In order to verify the validity of this proposal, simulations were carried out in R for a backcross type design of 100 progeny. There were six markers equally distributed on a 1 M linkage group. There was one QTL of 0.5σ of effect for which two true locations were envisaged: 0.1 M, i.e. between markers, and 0.2 M, i.e. on a marker. Table 7 shows the comparison of RMSE between "before" and "after" adjusting the estimated QTL location when the estimated QTL position was on one of the markers in the first place. The distributions of estimated QTL location before and after the adjustment of the estimated QTL location are presented in Figure 12. The RMSE was always smaller after the position had been adjusted, even when the true QTL location was in 0.2 M, i.e. on a marker location. In this example, the proportion of false QTL locations on the markers was effectively decreased by the proposed algorithm.

**Table 7:** RMSE of the QTL location before or after adjustment

	True QTL location (M)	
	
	0.1	0.2
Before^1^	28.6	23.6
After^2^	26.6	21.3

Results based on 5000 simulations per case in a backcross population of 100 progeny. There were 6 markers equally distributed on 1 M.

^1^RMSE for the estimated QTL location.

^2^RMSE for the estimated QTL location after adjusting the estimated QTL location by the proposed algorithm.

## Discussion

In order to study the elements that give rise to the bias on the estimated QTL position, we checked whether the distribution of the test statistic changed along the locations on the linkage group. More precisely, we checked if the significance threshold remained the same at a marker and at a non-marker location. Under the null hypothesis of "no QTL on the linkage group", the asymptotic distribution of the LRT at a given point is well known and identical for all locations. It will be getting closer to the central *χ*^2 ^distribution with a degree of freedom depending on the number of parameters fixed under the null hypothesis [20], i.e. here the number of sires or dams for which a QTL effect was estimated. Nevertheless, the population size is most often not large enough to make the LRT reach its asymptotic distribution for all the locations on the linkage group. The variability of the marker informativity along the linkage group may actually influence this convergence to asymptotic conditions, resulting in variability of the LRT distributions depending on the tested locations. Here, the differences between the empirical distributions of the LRT at each position along the linkage group were explored using a real example of an outbred type population. It appeared that the variability of informativity along the linkage group did not lead to a significative variability of the empirical distributions of the nominal test statistics. This observation is not contradictive to the bias on the estimated QTL location towards the locations of markers but it means that the bias is due to the process which defines the sup of LRT on the linkage group.

Some analytical results concerning the bias of the estimated QTL location were obtained in a backcross type population, i.e. a backcross between inbred lines. We identified a common source of bias under the "no QTL" and the "one QTL" hypotheses, and also showed the possible influence of several parameters under "one QTL" hypothesis, such as the population size, the marker density, the QTL effect and the true QTL location. Using simulations, we verified the existence of a bias on the estimation of the QTL location using the interval mapping method, under the null and the alternative hypotheses, when family structure are more complex than the backcross design considered by Walling *et al. *[4]. Simulations of outbred populations confirmed that this bias is influenced by the size of the population and the density of the genetic map, as well as by the QTL effect under the alternative hypothesis. We also demonstrated that the true QTL location, relatively to the flanking markers, had a significant impact on the accuracy of the estimated QTL location. Moreover, we quantified the bias of the estimated QTL location for various values of these parameters and validated the results by applying appropriate test statistics.

We showed that the population size does not affect the estimation of the QTL location under the null hypothesis. Under the alternative hypothesis, very similar values of RMSE or of proportion of QTL detected at marker locations were observed whatever *α*. On the other hand, a slight reduction in the bias seemed to be obtained when applying *α *< 0.01. However, the choice of a high significance level also implies a decrease of power and the detection of only few QTL. As a consequence, it cannot be considered an efficient way to correct the bias problem.

**Figure 13:**
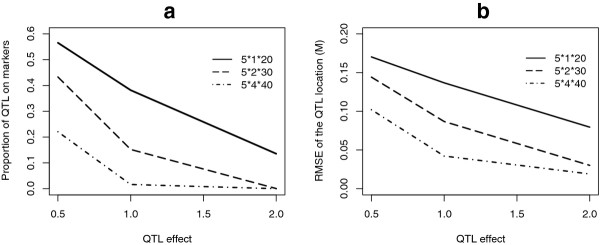
**Distribution of the bias depending on the population size and the QTL effect**. a. Proportion of estimated QTL locations on marker positions. b. RMSE of QTL location. Results are based on 5000 simulations per case. There were three markers at 0 M, 0.2 M and 0.4 M. The true QTL location was set at 0.1 M. 5*1*20 indicates that there were 5 sires, 1 dam per sire and 20 progeny per dam in the design.

Considering these results leads to a first recommendation which would be that the number of animals and/or markers must be adjusted to the desired test power and location accuracy. Figure 13 summarizes the variability of the bias in accordance with the population size and the QTL effect. The proportion of QTL mapped at a marker location and the RMSE of the QTL location present the same tendencies according to the variations in QTL effect and in population size. This confirms that the bias on the estimated QTL location is essentially due to the location of QTL on markers.

Secondly, concerning the particular case of eQTL detection, when the marker information is relatively sparse, for example when microsatellite markers are used for genotyping, it is necessary to measure several hundred of animals for transcriptomic data to obtain an accurate eQTL location. Finally, a population size of 300 progeny seems to be a good compromise in the detection of eQTL, even if only those which have relatively large effects will be detected.

Thirdly, it is clear that significant QTL detection located at a marker position should be considered with caution, especially when the population size, the marker density or the QTL effect are low. Hence, the approach proposed above is efficient to remedy the bias on the estimated QTL location in such situation.

## Conclusions

When we apply the interval mapping method on an outbred design to map QTL, the QTL is often incorrectly mapped at the position of a marker. In this work, this bias was studied by using analytical developments in backcross type population and simulated data in outbred populations. In the absence of QTL, adjusting the thresholds at the location of markers cannot reduce the bias, and the population size does not affect the bias. Under the hypothesis of having one QTL, the impact of some parameters on the bias was confirmed: when the population size and/or the QTL effect and/or the marker density are large enough, the bias is reduced. Moreover, the closer the QTL is to a marker location, the more accurate the estimation is. Therefore, caution should be taken when the QTL is mapped at a position of a marker, in particular for low power designs. In such cases, a method is proposed to correct the bias on the estimated QTL location. Simulations carried out in a backcross type population demonstrated that this method is valid to limit the bias.

## Methods

### Analyses on a real data set in pig

A real data set was used to illustrate some aspects of the present work. It is a porcine outbred population of 325 progeny issued from 4 sires. One example of eQTL analysis using the QTLMap software [21], i.e. the analysis of the chromosome SSC18 for 6 665 gene expression traits, was given. The linkage analysis method was applied according to Le Roy *et al. *[22] with a gene by gene procedure. For each gene, when the LRT was significant at the 5%_0 _level at the chromosome level, the estimated eQTL location was the location where the LRT was maximum on the linkage group.

The same familial structure was used to study the empirical distribution of the LRT along the linkage group. Two thousand simulations were performed under the null hypothesis of "no QTL" on the chromosome SSC1 which carried 16 microsatellite markers. A polygenic heritability coefficient of 0.5 was assumed for the trait (see http://www.inra.fr/qtlmap).

### Simulations of an Ornstein-Uhlenbeck process

The asymptotic distribution of the LRT process at marker positions was shown as being the square of an OU process [16]. Let's *X_t _*denote the value of this OU process at the *t *location. *X_t _*could be described as:

$$dX_{t}=2X_{t}dt+2dW_{t}$$

where *W_t _*denotes the Brownien movement.

In a backcross type population, the mean of this process is 0 and the autocovariance is: *cov*(*X_t_, X*_*t*'_) = *e*^-2|*t*-*t*'| ^with *t *and *t' *in the Haldane distance unit [16].

To simulate this process, we considered a linkage group with *mk *markers. We generated *mk *independent random numbers z_0_, z_1_, ..., z_*mk *_from a normal distribution with mean 0 and variance 1 with the function *rnorm*in R. We defined *X*_0 _= z_0_. Then, a discrete analog of the OU process [23] was given by:

$$X_{s}=e^{-2\tau }X_{s-1}+\sqrt{1-e^{-4\tau }}z_{s}$$

with *s *= 1, ..., *mk*, where *τ *denotes the spacing of two adjacent markers in Morgan. This sequence is a first-order autoregressive sequence.

### Simulations of outbred type population

The QTLMap software [21] was used to simulate and analyse the data sets. QTLMap allowed the simulation of complete experimental designs with pedigree, genetic map, genotypes and phenotypes http://www.inra.fr/qtlmap. The population structure was a mixture of full and half sib families for given numbers of sires (*s*), of dams per sire (*d*) and of progeny per dam (*p*). Most often, 3 markers were equally distributed on a 0.4 M linkage group. Each marker had 6 alleles with equal frequencies in the parental population. The QTL was simulated at 0.1 M and all sires and dams were heterozygous for the QTL. The phenotypes of the progeny were simulated as follows:

(5) $$y_{ijk}=u_{i}+u_{ij}+a\;g_{ijk}\left(t_{0}\right)+e_{ijk}$$

*y_ijk _*is the phenotype of the progeny *ijk *of the sire *i *and of the dam *ij. u_i _*and *u_ijk _*denote the polygenic effects, of the sire *i *and of the dam *ij *respectively, which follow a normal distribution with mean 0 and variance $\sigma_{u}^{2}$. *a *denotes the QTL allelic substitution effect and *g_ijk _*(*t*_0_) is the genotypic value of *ijk *at the QTL location *t*_0_. *g_ijk _*takes value 1, 0 or -1 depending onthe QTL genotype, *QQ, Qq *or *qq*, respectively. *e_ijk _*is a random normal variable with mean 0 and variance $\sigma_{e}^{2}$. The variance within QTL genotype is $\sigma^{2}=2\sigma_{u}^{2}+\sigma_{e}^{2}$ and *a *is expressed in σ unit. The heritability coefficient, equal to $4\sigma_{u}^{2}/\sigma^{2}$, was fixed at 0.25.

For each of the cases studied, the results were based on 5 000 simulations, either under the null hypothesis (*H*_0_: there is no QTL segregating on the linkage group, i.e. *a *= 0) or under the alternative hypothesis (*H*_0_: there is one QTL segregating on the linkage group, i.e. most often *a *= 1σ). For each simulated dataset, the estimated QTL position was the location of the linkage group where the LRT was maximum.

Under the null hypothesis, simulations were carried out so as to compare the influence of the population size with 6 levels: 60 (3*s*,1*d*,20*p*), 80 (4*s*,1*d*,20*p*), 100 (5*s*,1*d*,20*p*), 300 (5*s*,2*d*,30*p*), 400 (5*s*,2*d*,40*p*), 800 (5*s*,4*d*,40*p*) progeny. Under the H1 hypothesis, only 3 of these population sizes were considered: 100, 300 and 800 progeny.

To understand how the QTL effect affects the estimation of the QTL location, a population of 100 progeny was simulated with a QTL effect ranging from 0.5 σ to 4 σ.

Other simulations were performed in a population of 300 progeny. Firstly, to check the bias extent depending on the marker density, samples with 2, 3, 5, 7 or 11 markers equidistant in a linkage group of 0.6 M were simulated, under the null and under the alternative hypotheses. Under *H*_1_, one QTL was simulated at 0.25 M (*a *= 1σ). Secondly, to test how the true QTL location may affect the bias, we performed simulations under *H*_1 _with a QTL (*a *= 1σ) lying at 0 M, 0.05 M, 0.10 M, 0.15 M, 0.2 M on a linkage group of 0.4 M with two flanking markers at 0 M and 0.4 M.

### Criteria

In each of the cases studied, the empirical distribution of the estimated QTL location was obtained from the 5000 locations of maximum of LRT of the simulations. The proportion of simulations for which the QTL location was estimated at one marker position was retained to quantify the variability of the bias depending on the parameters. Under the alternative hypothesis, beside the proportion of the QTL which co-localized with a marker, the root mean squared error (RMSE) of the QTL location was chosen to describe the bias variability which depends on the parameters. What is more, the power of the QTL detection was calculated for a first type error *α *= 0.01 and 0.05. For all simulations and for simulations with a significant QTL at the level *α *= 0.01 or 0.05, the proportion of QTL that were estimated at a marker location and the RMSE of the estimated QTL location were computed. The RMSE computation was given by the following formula

$$\text{RMSE}=\sqrt{\frac{1}{L}\overset{L}{\underset{l=1}{\sum }}\;{\left({\hat{t}}_{l}-t_{0}\right)}^{2}},$$

where *L *is the number of simulations (all, significant at the level 0.05 or at the level 0.01), ${\hat{t}}_{l}$ is the *l^th ^*estimated QTL position and *t_0 _*is the true QTL position.

### Hypothesis test

Appropriate statistical tests are needed to evaluate which parameters affect the bias of the estimated QTL position. ANOVA was not adequate to test the equality of the average QTL position in two different conditions (e.g. 2 population sizes) because of the non normality of the QTL position estimator. Therefore, two nonparametric tests were combined in order to test which parameters affect the bias, and how they influence the variation of the QTL location estimation. This was performed in two steps: (1) the parameters which influence the accuracy of the estimated QTL location were identified. This step was carried out with a Kolmogorov-Smirnov test; (2) for the parameters identified in the first step, a description of their effect on the accuracy of the estimated QTL position was made. This step was performed with a Mann-Whitney-Wilcoxon test [24].

1. Kolmogorov-Smirnov test (KS): this test was applied in order to check whether a parameter affected the estimation of the QTL position. For each value of the parameter, an empirical distribution of the estimated QTL location was obtained using 5 000 simulations. The two hypotheses compared by the KS test were:

$$\text{H}0:\;F_{a}=F_{b}\;\;\text{H}1:\;F_{a}\neq F_{b},$$

where *F_a_, F_b _*denote the distribution of the estimated QTL position under the conditions *a *and *b*, respectively. For a given parameter, all the distributions were compared by pairs with the function *ks.test *in R. If all pair comparisons concluded to accept the null hypothesis, it means that the value of this parameter did not influence the estimation of the QTL position.

Mann-Whitney-Wilcoxon test (MWW): when the null hypothesis in the first step was rejected, the MWW test was used to understand how the parameter affected the estimation with the function *wilcox.test *in R. The hypotheses compared were:

$$\text{H}0:\;D_{a}=D_{b}\;\;\text{H}1:\;D_{a}\leq D_{b},$$

where *D_a_, D_b _*denote the absolute values of the deviations between the estimated QTL position and the assumed, i.e. the true position, under the condition *a *and *b*, respectively. A smaller median *D *corresponds to a more accurate position estimation.

## Appendix

### Appendix I

Let us denote *M^k ^*the genotype of the markers at 0 and *T *for individual *k *and *p_kt _*= ℙ (*g_k_*(*t*) = 1|*M^k^*). Then using a linearized likelihood function instead of the mixture of two normal distributions, the LRT can be written as: $\begin{array}{cc} LRT\left(t\right) & =-2\ln\frac{L\left(y_{1},\ldots ,y_{n}\right)}{\max_{a}L\left(y_{1},\ldots ,y_{n};a\right)}\\ & \approx -2\ln\frac{\prod \phi \left(y_{k};0,1\right)}{\max_{a}\prod \phi \left(y_{k}+\frac{a}{2}\left(1-2p_{kt}\right);0,1\right)}\\ & =\frac{{\left[\sum y_{k}(1-2p_{kt})\right]}^{2}}{\sum {\left(1-2p_{kt}\right)}^{2}}, \end{array}$

where $\phi \left(x;\mu ,\sigma \right)=\frac{1}{\sqrt{2\pi }\sigma }e^{-\frac{{\left(x-\mu \right)}^{2}}{2\sigma^{2}}}$ and the distribution of *p_kt _*is given as:

**Table:** 

*p_kt_*	Probability	*M^k^*
(1-*θ_t_*)(1-*θ_T-t_*)/(1-*θ_T_*)	(1-*θ_T_*)/2	*M*_1_*M*_1_*M*_2_*M*_2_

(1-*θ_t_*)*θ_T-t_/θ_T_*	*θ_T_*/2	*M*_1_*M*_1_*M*_2_*m*_2_

*θ_T-t_θ_t_/θ_T_*	(1-*θ_T_*)/2	*M*_1_*m*_1_*M*_2_*m*_2_

*θ_t_*(1-*θ_T-t_*)/*θ_T_*	*θ_T_*/2	*M*_1_*m*_1_*M*_2_*M*_2_

Note that *x_k_*(*t*) = **E**(*g_k_*(*t*)|*M^k^*) = 2*p_kt _*- 1, so the LRT at each position *t *can be described as $LRT\left(t\right)=\frac{{\left[\sum y_{k}\;x_{k}\left(t\right)\right]}^{2}}{\sum x_{k}^{2}\left(t\right)},$ and the distribution of *x_k _*(*t*) for each individual *k *is

**Table:** 

*x_k _*(*t*)	Probability	*M^k^*
(1-*θ_t_*-*θ_T-t_*)/(1-*θ_T_*)	(1-*θ_T_*)/2	*M*_1_*M*_1_*M*_2_*M*_2_

(*θ_T-t_*-*θ_t_*)/*θ_T_*	*θ_T_*/2	*M*_1_*M*_1_*M*_2_*m*_2_

*(θ_t_*+*θ_T-t_-*1)/(1-*θ_T_*)	(1-*θ_T_*)/2	*M*_1_*m*_1_*M*_2_*m*_2_

(*θ_t_*-*θ_T-t_*)/*θ_T_*	*θ_T_*/2	*M*_1_*m*_1_*M*_2_*M*_2_

### Appendix II

Replacing $y_{k}=\frac{a}{2}x_{k}\left(t_{0}\right)+\varepsilon_{k}$ in *LRT *(3), we have $\begin{array}{cc} LRT\left(t\right) & =\frac{{\left[\sum \left(\frac{a}{2}x_{k}\left(t_{0}\right)+\varepsilon_{k}\right)x_{k}\left(t\right)\right]}^{2}}{\sum x_{k}^{2}\left(t\right)}\\ & =\frac{1}{4}a^{2}\frac{{\left[\sum x_{k}\left(t_{0}\right)x_{k}\left(t\right)\right]}^{2}}{\sum x_{k}^{2}\left(t\right)}+\frac{{\left[\sum \varepsilon_{k}x_{k}\left(t\right)\right]}^{2}}{\sum x_{k}^{2}\left(t\right)}+a\frac{\sum x_{k}\left(t_{0}\right)x_{k}\left(t\right)}{\sum x_{k}^{2}\left(t\right)}\sum \varepsilon_{k}x_{k}\left(t\right)\\ & =f\left(t\right)+\varepsilon_{1}\left(t\right)+\varepsilon_{2}\left(t\right), \end{array}$

where in the case of large sample, we have

• $f\left(t\right)=\frac{1}{4}a^{2}\frac{{\left[\sum x_{k}\left(t_{0}\right)x_{k}\left(t\right)\right]}^{2}}{\sum x_{k}^{2}\left(t\right)}$ and $f\left(t\right)/n\to \frac{1}{4}a^{2}Var\left(x\left(t_{0}\right)\right)\rho_{tt_{0}}^{2}$ when *n *→ ∞ according to the law of large numbers.

• $\varepsilon_{1}\left(t\right)=\frac{{\left[\sum \varepsilon_{k}x_{k}\left(t\right)\right]}^{2}}{\sum x_{k}^{2}\left(t\right)}$ is the LRT under the no QTL hypothesis.

• $\varepsilon_{2}\left(t\right)=a\frac{\sum x_{k}\left(t_{0}\right)x_{k}\left(t\right)}{\sum x_{k}^{2}\left(t\right)}\sum \varepsilon_{k}x_{k}\left(t\right)\sim a\frac{Cov\left(x\left(t\right),x\left(t_{0}\right)\right)}{Var\left(x\left(t\right)\right)}\sum \varepsilon_{k}x_{k}\left(t\right)$ is a residual error, linear combination of gaussian random variables. Its distribution is approximated as $N\left(0,na^{2}Var\left(x\left(t\right)\right)\rho_{tt_{0}}^{2}\right).$

### Appendix III

Considering the two first terms in the expression of $\frac{1}{n}LRT\left(t\right)$ (4), when *n *tends to infinity, $\frac{1}{n}\varepsilon_{1}\left(t\right)$ will converge to 0 at each position *t*. So the amplitude of the curve representing the term $\frac{1}{n}\varepsilon_{1}\left(t\right)$, with respect to that of the first term, is reduced. In the same way, as *a*^2 ^increases, the amplitude of the first term becomes larger with respect to that of the second term.

The proof of the influence of *t*_0 _and *T *will use the results in this following lemma:

**Lemma 1**. Given two markers at the location 0 and *T *in a linkage group of length *T *and assuming a QTL located at *t*_0_, from the distribution of *Var(x(t)) *and applying the Taylor series expansion in case of small T, we have:

1. $Var\left(x\left(t_{0}\right)\right)=\frac{{\left(1-\theta_{t_{0}}-\theta_{T-t_{0}}\right)}^{2}}{1-\theta_{T}}+\frac{{\left(\theta_{t_{0}}-\theta_{T-t_{0}}\right)}^{2}}{\theta_{T}}\approx \frac{4}{T}t_{0}^{2}-4t_{0}+1.$

2. $Cov\left(x\left(t\right),x\left(t_{0}\right)\right)=\frac{\left(1-\theta_{t}-\theta_{T-t}\right)\left(1-\theta_{t_{0}}-\theta_{T-t_{0}}\right)}{1-\theta_{T}}+\frac{\left(\theta_{t}-\theta_{T-t}\right)\left(\theta_{t_{0}}-\theta_{T-t_{0}}\right)}{\theta_{T}}\approx \frac{4}{T}tt_{0}-2\left(t+t_{0}\right)+1.$

Now let us assume *T *< 1 and consider the peak-to-peak amplitude of the first term of $\frac{1}{n}LRT\left(t\right)$, $g\left(t\right)=\frac{1}{4}a^{2}Var\left(x\left(t_{0}\right)\right)\rho_{tt_{0}}^{2}$, i.e., the deviation between highest amplitude value and lowest amplitude value:

$$\delta =\underset{t\in \left[0,T\right]}{\max}g\left(t\right)-\underset{t\in \left[0,T\right]}{\min}g\left(t\right).$$

If $t_{0}\in \left[\frac{T}{2},T\right]$, then the max_*t*∈[0, *T*]_*g*(*t*) is reached at *t *= *t*_0 _and the min_*t*∈[0, *T*]_*g*(*t*) is reached at 0. Hence, we have:

$$\begin{array}{cc} \delta & =\frac{a^{2}}{4}\left[Var\left(x\left(t_{0}\right)\right)-Cov^{2}\left(x\left(0\right),x\left(t_{0}\right)\right)\right]\\ & \approx a^{2}\left(\frac{1}{T}-1\right)t_{0}^{2}. \end{array}$$

It can be seen that when *t*_0 _→ *T *from $\frac{T}{2}$ and/or when *T *decreases, *δ *will become larger.

If $t_{0}\in \left[0,\frac{T}{2}\right]$, then the max_*t*∈[0, *T*]_*g*(*t*) is reached at *t *= *t*_0 _and the min_*t*∈[0, *T*]_*g*(*t*) is reached at *T*. Hence, we have:

$$\begin{array}{cc} \delta & =\frac{a^{2}}{4}\left[Var\left(x\left(t_{0}\right)\right)-Cov^{2}\left(x\left(T\right),x\left(t_{0}\right)\right)\right]\\ & \approx a^{2}\left(\frac{1}{T}-1\right){\left(t_{0}-T\right)}^{2}. \end{array}$$

It can be seen that when *t*_0 _→ 0 from $\frac{T}{2}$, *δ *will become larger. Likewise, we set a constant *c *for the distance between the argmax_*t*∈[0, *T*]_*g*(*t*) and argmin_*t*∈[0, *T*]_*g*(*t*). Then, when *T *changes, the position of QTL is *t*_0 _= *T -c *and

$$\delta \approx a^{2}\left(\frac{1}{T}-1\right)c^{2}.$$

Therefore, when *T *becomes larger, δ decreases.

In conclusion, the amplitude of *g(t) *will be greater as the QTL position tends to one marker and/or the distance between the markers decreases.

## Competing interests

The authors declare that they have no competing interests.

## Authors' contributions

PLR conceived this study. XW carried out the study and drafted the manuscript, supervised by PLR and JME. XW, HG, CM and OF wrote updated versions of the QTLMap software. All authors read and revised and approved the final manuscript.
